# Prediction of Synergistic Antibiotic Combinations by Graph Learning

**DOI:** 10.3389/fphar.2022.849006

**Published:** 2022-03-08

**Authors:** Ji Lv, Guixia Liu, Yuan Ju, Ying Sun, Weiying Guo

**Affiliations:** ^1^ College of Computer Science and Technology, Jilin University, Changchun, China; ^2^ Key Laboratory of Symbolic Computation and Knowledge Engineering of Ministry of Education, Jilin University, Changchun, China; ^3^ Sichuan University Library, Sichuan University, Chengdu, China; ^4^ Department of Respiratory Medicine, The First Hospital of Jilin University, Changchun, China; ^5^ The First Hospital of Jilin University, Changchun, China

**Keywords:** antibiotic combination, antimicrobial resistance, graph learning, bacterial infection, synergy effect

## Abstract

Antibiotic resistance is a major public health concern. Antibiotic combinations, offering better efficacy at lower doses, are a useful way to handle this problem. However, it is difficult for us to find effective antibiotic combinations in the vast chemical space. Herein, we propose a graph learning framework to predict synergistic antibiotic combinations. In this model, a network proximity method combined with network propagation was used to quantify the relationships of drug pairs, and we found that synergistic antibiotic combinations tend to have smaller network proximity. Therefore, network proximity can be used for building an affinity matrix. Subsequently, the affinity matrix was fed into a graph regularization model to predict potential synergistic antibiotic combinations. Compared with existing methods, our model shows a better performance in the prediction of synergistic antibiotic combinations and interpretability.

## Introduction

Antibiotic resistance is a growing health crisis, and it is emerging globally (Author Anonymous, 2013; Zhabiz et al., 2014; Murray et al., 2022). This crisis has been ascribed to the wide use and even abuse of antibiotics in the clinic, as well as a lack of economic incentives and market regulation of new antibiotic development (Ventola, 2015; Farha et al., 2021). An increasing number of Big Pharma have stopped developing new antibiotics, and the number of new FDA-approved antibiotics has gradually decreased since the 1980s (Ventola, 2015). Therefore, we have to find an alternative way to address this pressing public health problem.

Antibiotic combinations offer an effective strategy to combat antibiotic resistance (Tyers and Wright, 2019; Lv et al., 2021). Generally, antibiotic combinations can be divided into three groups: synergy, additive, and antagonism (Cokol et al., 2011). Synergistic antibiotic combinations are often used in clinics because they can offer better efficacy at lower doses (Mathers, 2015). In the microbiology laboratory, synergy or antagonism is usually identified through the fractional inhibitory concentration index (FICI) (Odds, 2003). However, this approach is expensive and time-consuming. To date, more than 300 antibiotics have been discovered (Wright, 2014), generating at least 44, 850 drug pairs. In addition, the efficacies of antibiotic combinations were also affected by doses (Maan et al., 2021), metabolic conditions (Cokol et al., 2018), and bacterial strains (Chandrasekaran et al., 2016). Consequently, millions of drug pairs need to be tested. As a result, it is impossible to screen synergistic antibiotic combinations by experimental approaches. Recently, with the development of artificial intelligence, many researchers have started to use computational approaches to identify synergistic drug combinations (Sheng et al., 2017; Weinstein et al., 2017). They used drug structures (Mason et al., 2017; Mason et al., 2018) and chemo-genomics data (Chandrasekaran et al., 2016) as input to the “black-box” machine learning model to predict potential synergistic drug combinations. Although these models have shown good performance (Chandrasekaran et al., 2016; Mason et al., 2017; Mason et al., 2018), some limitations still exist. First and foremost, the curse of dimensionality is a serious problem. Specifically, the number of features (chemogenomic data: 3,979 and Morgan fingerprint: 2048) is much greater than the number of training sets (approximately 100). Furthermore, some features [e.g., chemo-genomics (Nichols et al., 2011)] are not only difficult to obtain but also hard to use to explain the mechanisms of the synergy effect. Therefore, more effective and interpretable features are needed.

**FIGURE 1:**
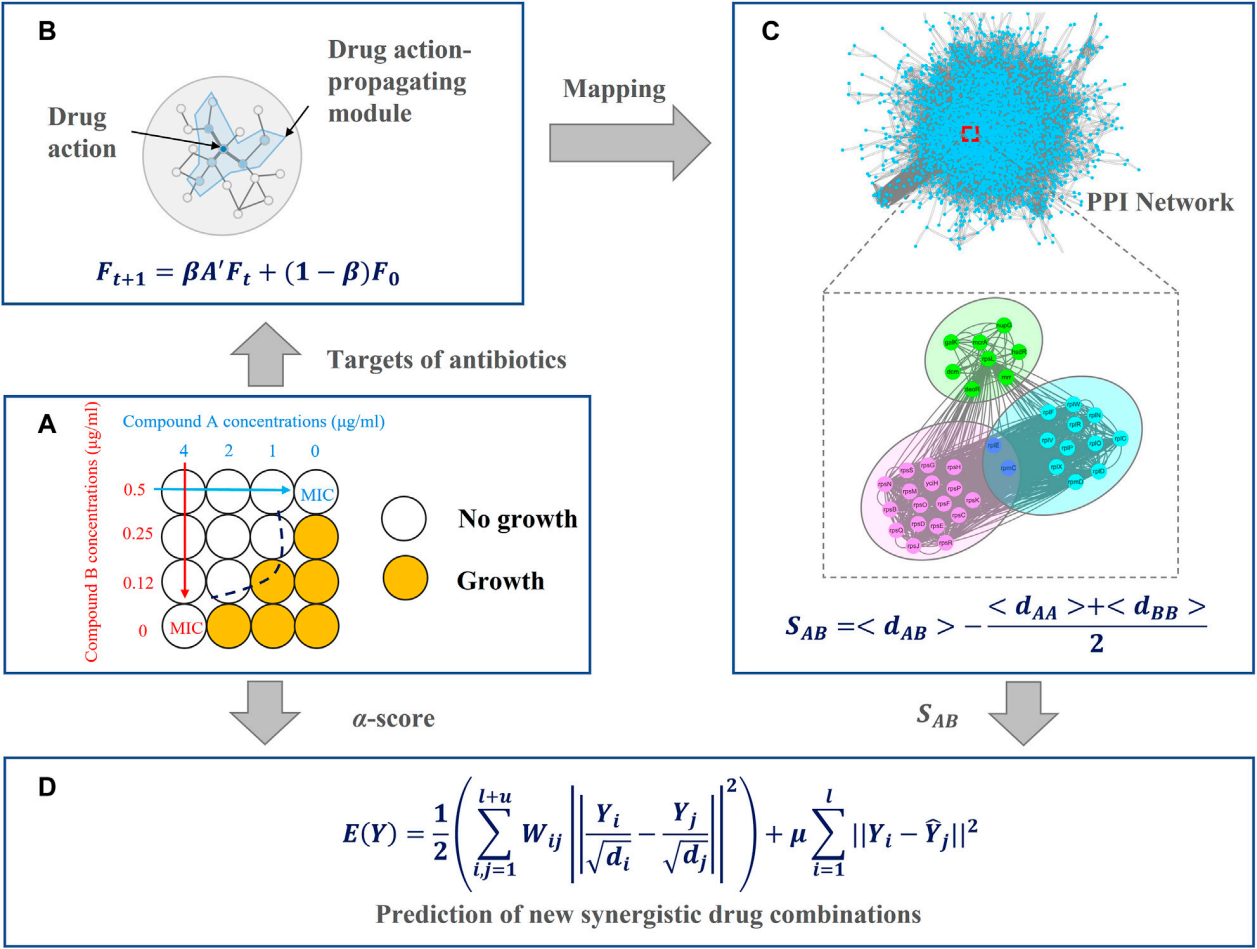
Overview of the network-based method for antibiotic combinations, including four main parts **(A)** collect antibiotic combinations and target information from the literature **(B)** describe drug actions by network propagation **(C)** evaluate relationships between each drug pair by network proximity, and **(D)** predict new synergistic antibiotic combinations.

Network pharmacology is a new paradigm for drug discovery (Hopkins, 2008) that can help us better understand intricate relationships between drugs, targets, pathways, and diseases (Menche et al., 2015; Cheng et al., 2019; Wang J. et al., 2021; Wang Y. et al., 2021; Li et al., 2021). In network pharmacology, the actions of drugs are regarded as perturbations to the network (Csermely et al., 2013). When a node is perturbed, neighboring nodes will also be affected (Saraswathi et al., 2009). However, perturbation experiments are expensive and time-consuming (Nichols et al., 2011). In this study, we introduced a network propagation method to simulate perturbation patterns of drug pairs (Figure 1B). Intuitively, variations in the medication regimen (synergy or antagonism) cause them to have a slight difference in the network structure and dynamics. Subsequently, we used the network proximity method (Figure 1C) to quantify the relationships between the interactomes between targets of different drugs. We found that synergistic antibiotic combinations tend to have smaller network proximity. In other words, network proximity is a good parameter to classify drug pairs and to avoid the curse of dimensionality. Finally, we introduced a mechanism-driven graph regularization model to predict synergistic antibiotic combinations based on this finding (Figure 1D). The results demonstrated that our method outperformed other existing methods in the prediction of synergistic antibiotic combinations and interpretability.

## Materials and Methods

In this section, we introduced the architecture of our model, as shown in Figure 1. In Figure 1A, we collected antibiotic combinations and their targets from the literature. In Figure 1B, the targets of these antibiotics were fed into the network propagation model. When a node is perturbed, neighboring nodes will also be affected, resulting in a subnetwork. We named this subnetwork as a drug action-propagating module (DAPM). In Figure 1C, we used a network proximity model to quantify the relationships between drug pairs. In Figure 1D, the network proximity of each drug pair was converted to an affinity matrix. This affinity matrix and the known antibiotic combinations were employed to build a graph regularization model, thereby predicting new synergistic antibiotic combinations.

### Constructing the Protein–Protein Network and Drug–Target Network

We constructed the PPI network of *Escherichia coli* based on the STRING database version 11.5 (Szklarczyk et al., 2020). The interactions with a score less than 0.7 were ignored. The ultimate network included 59, 496 interactions involving 4, 020 proteins.

We collected drug–target interactions from previous literature reports or the DrugBank database (Wishart et al., 2017). Since we used the data from the *in vitro* antimicrobial test, proteins from bacteria were considered and proteins of *Homo sapiens* were ignored in this study.

### Collecting Pairwise Antibiotic Combinations

In this study, we focused on pairwise antibiotic combinations by recent experimental data of the *Escherichia coli* strain MG1655 (Chandrasekaran et al., 2016). Interactions were quantified based on the α-score, and the following three types were used: synergy (α-score ≤ −0.25), additive (−0.25< α-score < 1), and antagonism (α-score ≥ 1) (Cokol et al., 2011). In this study, we only considered antibiotics with known targets (protein or RNA). In total, 91 pairwise antibiotic combinations involving 14 antibiotics were retained.

### Network Propagation of Drug Action

Targets of the aforementioned antibiotics were fed into a network propagation model (Vanunu et al., 2010) to simulate the propagation of drug-action effects on the PPI network:

$$F_{t+1}=\beta A^{\prime }F_{t}+\left(1-\beta \right)F_{0},$$ (1)

where β is a parameter ( 0≤β≤1 ). Vanunu et al., (2010) confirmed that the algorithm is not sensitive to the choice of β as long as it is above 0.5, so we set β to 0.7. $A^{\text{'}}=D^{-1/2}AD^{-1/2}$ in which *D* is a diagonal matrix, and the values of the diagonal element (*d*_*ii*_) are equal to the degree of the vertexes (*k*_*i*_), and *A* is an adjacency matrix. *F*_*0*_ denotes a matrix, in which “1” indicates that the drug can bind to this target, and if the drug can bind multiple targets (*n*), the values were set as 1/*n*. During each iteration, nodes can not only receive the information from their neighbors (first term) but can also retain their initial information (second term), resulting in a DAPM. Next, let us show that formula (1) converges. The general term formula of formula (1) is

$$F_{t}={\left(\beta A^{\prime }\right)}^{t}F_{0}+\left(1-\beta \right)\overset{t-1}{\underset{i=0}{\sum }}{\left(\beta A^{\prime }\right)}^{i}F_{0}$$ (2)

Since 0<β<1 and the eigenvalues of $A^{\text{'}}$ are in the range of −1 to 1 (according to the Perron–Frobenius theorem),

$$\underset{\text{t}\to \infty }{\lim}{\left(\beta A^{\prime }\right)}^{t}=0\;\text{and}\;\underset{\text{t}\to \infty }{\lim}\overset{\text{t}-1}{\underset{\text{i}=0}{\sum }}{\left(\beta A^{\prime }\right)}^{i}={\left(I-\beta A^{\prime }\right)}^{-1}$$ (3)

Hence,

$$\underset{t\to \infty }{\lim}F_{t}={\left(I-\beta A^{\prime }\right)}^{-1}$$ (4)

### Quantification of Relationships Between Each Drug Pair

Subsequently, the Jaccard index (Eq. 5) and network proximity model (Eq. 6) (Menche et al., 2015; Cheng et al., 2019) were used to quantify the relationships of each DAPM:

$$J_{AB}=\frac{\left|S_{A}\cap S_{B}\right|}{\left|S_{A}\cup S_{B}\right|}$$ (5)

where *S*_*A*_ and *S*_*B*_ are the nodes of drug A and drug B in their DAPMs, respectively.

$$S_{AB}\equiv <d_{AB}>-\frac{<d_{AA}>+<d_{BB}>}{2}$$ (6)

where $<d_{AA}>$ and $<d_{BB}>$ are the mean shortest distances between each pair of nodes in the DAPM. $<d_{AB}>$ is the mean shortest distance between each pair of nodes between the DAPM of drug *A* and the DAPM of drug *B*:

$$<d_{AB}>=\frac{1}{\left||A||+||B|\right|}\underset{y\in A}{\sum }min_{x\in B}d\left(x,y\right)$$ (7)

where *A* and *B* are the DAPMs of drug *A* and drug *B*, respectively. d(x,y) is the shortest distance between node *x* and node *y*. In the next section, we demonstrated how to build the affinity matrix and graph regularization model based on network proximity.

### Prediction of Synergistic Antibiotic Combinations Based on Graph Regularization

Given three drugs (drug A, drug B, and drug C), if drug A–drug B is a synergistic antibiotic combination and drug A and drug C are pharmacologically similar, then drug C–drug B will likely be a synergistic antibiotic combination. Therefore, we can define a loss function:

$$E\left(Y\right)=\frac{1}{2}\left(\overset{l+u}{\underset{i,j=1}{\sum }}W_{ij}||\frac{Y_{i}}{\sqrt{d_{i}}}-\frac{Y_{j}}{\sqrt{d_{j}}}|{|}^{2}\right)+\mu \overset{l}{\underset{i=1}{\sum }}||Y_{i}-{\hat{Y}}_{i}|{|}^{2}$$ (8)

where *Y* and $\hat{Y}$ are the entire and the known drug combination matrix, respectively. μ ( μ>0 ) is the regularization parameter. *d*_*i*_ is the degree of node *i*. The key to this model rests on the construction of the affinity matrix *W*, which is calculated by Eq. 9.

$$W_{ij}=\{\begin{array}{cc} 1, & \;-1<S_{ij}<0;\\ 0, & \;otherwise \end{array}$$ (9)

where *S*_*ij*_ is the network proximity (Eq. 6) between drug *i* and drug *j*. The classifying model is as follows.

$$Y^{*}=argmin\;E\left(Y\right)$$ (10)

We then take the derivation of E(Y) with respect to *Y* to solve the optimization problem.

$${\frac{\partial E\left(Y\right)}{\partial Y}|}_{Y=Y^{*}}=Y^{*}-QY^{*}+\mu \left(Y^{*}-\hat{Y}\right)=0$$ (11)

where $Q=D^{-1/2}WD^{-1/2}$ . The detailed derivation of Eq. 11 can be found in the supporting information, and the analytical solution of Eq. 11 is

$$Y^{*}=\delta {\left(I-\gamma Q\right)}^{-1}\hat{Y}$$ (12)

where *I* is the identity matrix, γ=11+μ , and δ=μ1+μ .

### Performance Evaluation Metrics

The performance of the graph regularization model was estimated using the precision (Eq. 13), recall (Eq. 14), accuracy (Eq. 15), and F1 (Eq. 16), and these evaluation metrics can be defended as follows:

$$precision=\frac{TP}{TP+FP}$$ (13)

$$recall=\frac{TP}{TP+FN}$$ (14)

$$accuracy=\frac{TP+TN}{TP+FP+TN+FN}$$ (15)

$$F1=\frac{2\times precision\times recall}{precision+recall}$$ (16)

where TP, FP, FN, and TN are true positive, false positive, false negative, and true negative, respectively.

## Results

### The Data Set of Antibiotic Combinations

We used previously reported antibiotic combinations involving 14 antibiotics (Chandrasekaran et al., 2016) listed in Table 1. These antibiotics range over various mechanisms of action, including protein biosynthesis, DNA and RNA replication, folate metabolism, and cell wall biosynthesis. Since we concentrated on the subtle differences among synergy, additive, and antagonism, all 91 pairwise combinations fall into three categories, according to the α-score (Supplementary Table S1). Targets of these antibiotics were collected from previous literature studies (Pongs et al., 1973; Shen and Pernet, 1985; Buck and Cooperman, 1990; Pan et al., 1996; Onodera et al., 2002; Aracena et al., 2014; Kocaoglu and Carlson, 2015; Wekselman et al., 2017; Lin et al., 2018; Salehi et al., 2020; Wróbel et al., 2020). Because some antibiotics are RNA-targeted small molecules, ribosomal proteins that affect antibiotic binding are considered targets of antibiotics. For example, 30S ribosomal proteins S7 (rpsG) and S14 (rpsN) were shown to be the most important for tetracycline binding (Buck and Cooperman, 1990). Mutations of 50S ribosomal proteins L22 (rplV) and L4 (rplD) will lead to macrolide (erythromycin, etc.) resistance (Wekselman et al., 2017).

**TABLE 1:** List of antibiotics used for network analysis and their targets and mechanisms.

Drug	Abbreviation	Targets	Mechanism of action
Amikacin	AMK	rpsL Lin et al. (2018)	Protein synthesis, 30 S inhibition
Gentamicin	GEN	rpsL Lin et al. (2018)	Protein synthesis, 30 S inhibition
Tobramycin	TOB	rpsL Lin et al. (2018)	Protein synthesis, 30 S inhibition
Tetracycline	TET	rpsG, rpsN Buck and Cooperman (1990)	Protein synthesis, 30 S inhibition
Clarithromycin	CLA	rplD, rplV Salehi et al. (2020)	Protein synthesis, 50 S inhibition
Erythromycin	ERY	rplD, rplV Wekselman et al. (2017)	Protein synthesis, 50 S inhibition
Chloramphenicol	CHL	rplP Pongs et al. (1973)	Protein synthesis, 50 S inhibition
Ciprofloxacin	CIP	gyrA, parC Pan et al. (1996)	DNA gyrase inhibition
Levofloxacin	LEV	gyrA, parC Onodera et al. (2002)	DNA gyrase inhibition
Nalidixic acid	NAL	gyrA Shen and Pernet (1985)	DNA gyrase inhibition
Trimethoprim	TRI	folA Wróbel et al. (2020)	Folic acid biosynthesis inhibition
Oxacillin	OXA	dacB, ftsI Kocaoglu and Carlson (2015)	Cell wall
Cefoxitin	CEF	mrcA, mrcB, dacB, dacA, dacC, pbpG, ftsI Kocaoglu and Carlson (2015)	Cell wall
Nitrofurantoin	NIT	nfsA Aracena et al. (2014)	Multiple mechanisms

Network analysis showed that the shortest distance between targets of antibiotic combinations ranged from 0 to 5 (Supplementary Figure S1). Most antibiotic combinations (92.3%) did not share the same targets. Approximately thirty percent of antibiotic combinations were adjacent, and almost half of synergistic antibiotic combinations (57.1%) were included (Supplementary Figure S1). However, a considerable portion of antagonistic or additive antibiotic combinations have adjacent targets, but they are not synergistic (Supplementary Figure S1). Therefore, mere knowledge of the network structure may not be sufficient to explain the intricate interactions among antibiotic combinations and their targets. To investigate the network-based relationship between antibiotic combinations and their targets, we introduced network propagation (Vanunu et al., 2010) to predict the effect of antibiotics and antibiotic combinations on the PPI network.

### Network Propagation of Drug Actions

Network propagation has been used to quantify the influence of mutations in colorectal tumorigenesis (Shin et al., 2017). When a mutation arises in a node, perturbation spreads out along the protein–protein interaction (PPI) network and eventually forms a mutation-propagating module. Similar to mutation, if a drug acts on a node, neighboring nodes are also affected (Figure 1B) (Saraswathi et al., 2009). Predictably, the impact is greatest in its neighbors, whereas nodes far away from targets receive attenuated influences. Therefore, we can generate a subnetwork with drug targets as hubs, and the nodes ( $F_{i}^{\text{*}}\geq 0.0065$ ) will be incorporated into the subnetwork.

Based on the network propagation method (Eq. 1), these antibiotics and antibiotic combinations were mapped to the PPI network to investigate the potential relationships of these subnetworks (Figure 1). On average, DAPMs include approximately 13 nodes, although almost all drugs only have 1 to 2 targets. Because of the high threshold, each DAPM consisted almost exclusively of nearest neighbors. Interestingly, we found that there are areas of overlap for some antibiotic combinations and that antibiotic combinations are associated with the synergy effect (Figure 1C). Hence, we inferred that the structure of DAPKs can be used to quantify interactions between drug pairs and thereby predict synergetic antibiotic combinations.

### Network-Based Relationship Between DAMPs

**FIGURE 2:**
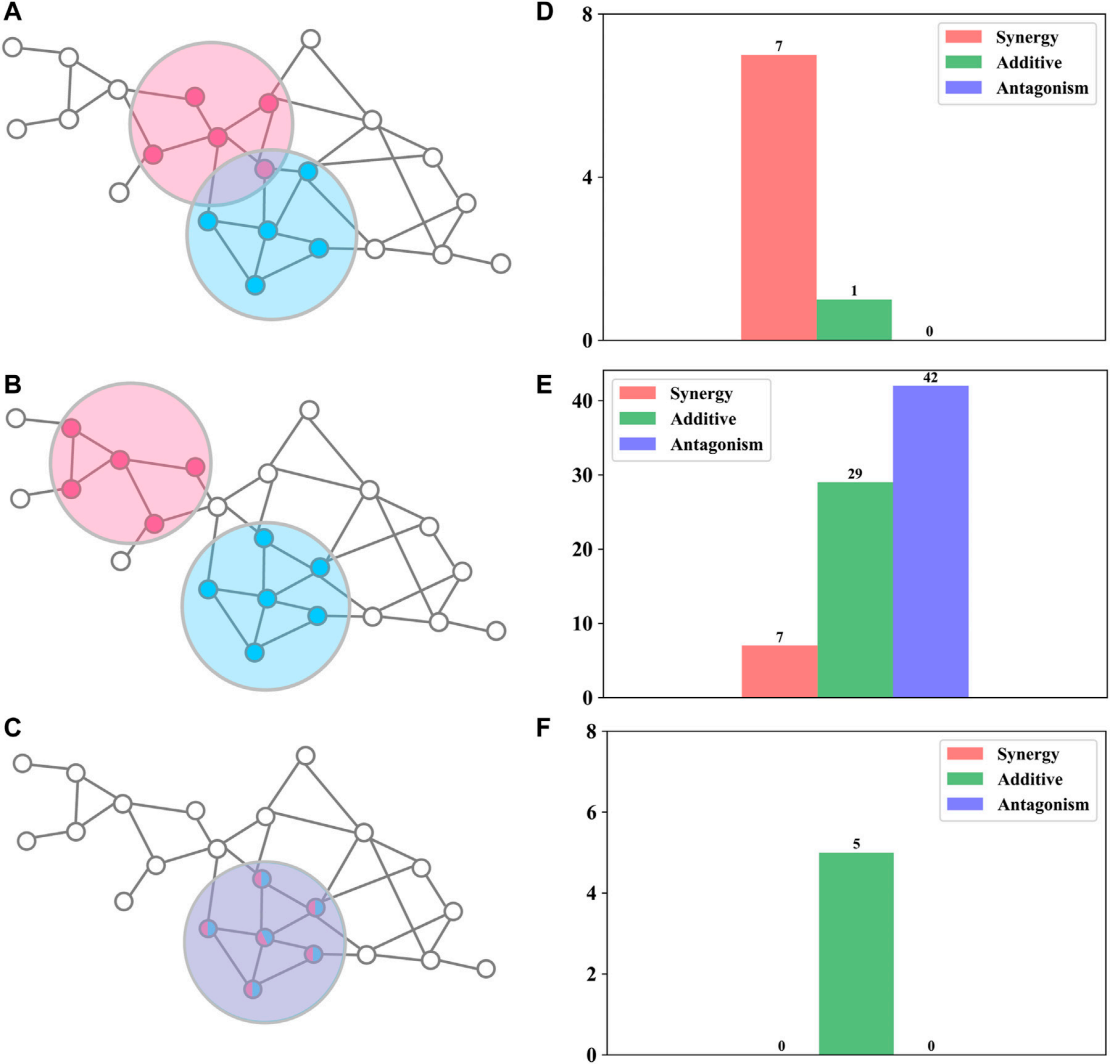
Relationships between drug interactions and network structures **(A–C)** Sketch map of the three topologically distinct classes **(D–F)** The number of synergistic, additive, and antagonistic drug combinations for the corresponding network structure.

Network proximity was used to investigate FDA-approved drug combinations (Cheng et al., 2019) and herb combinations in traditional Chinese medicine (Wang Y. et al., 2021; Zhang et al., 2021). Compared with random herd pairs, herd pairs in traditional Chinese medicine formulas tend to have smaller network proximity (Wang Y. et al., 2021). To probe whether it could also be used to distinguish synergy, additive, and antagonism, we used the Jaccard index (Eq. 5) and network proximity (Eq. 6) to quantify DAMP–DAMP interactions. We found that all possible antibiotic combinations can be divided into three topologically distinct categories: a) overlap: two DAMPs overlap but do not equate (Figure 2A); b) separation: two DAMPs are topologically separated (Figure 2B); and c) identical: two DAMPs are completely consistent (Figure 2C).

**FIGURE 3:**
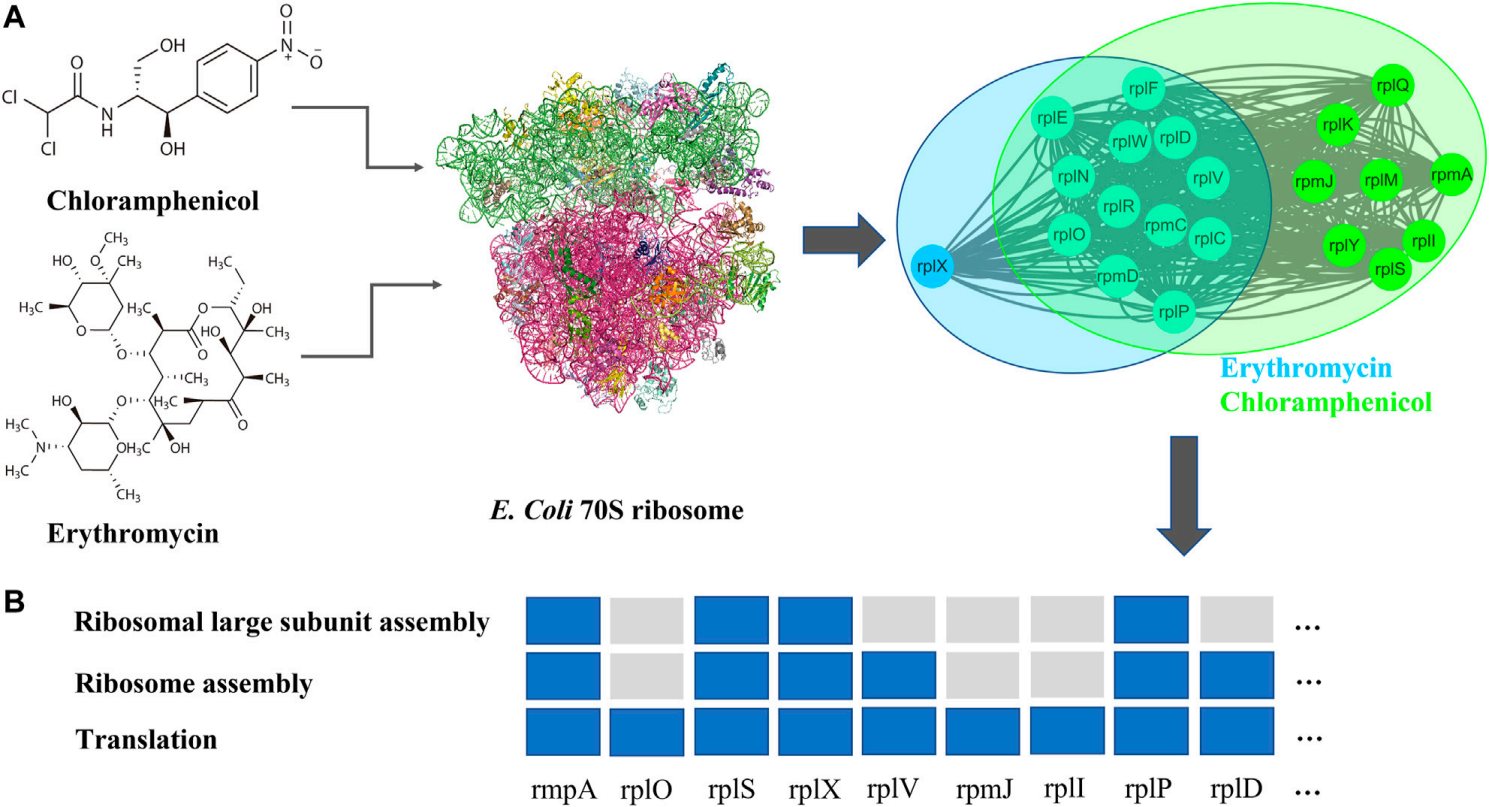
**(A)** Chemistry structural formula, targets (PDB ID: 4V48), and DAPMs of chloramphenicol and erythromycin **(B)** gene enrichment analysis (Mi et al., 2018) for DAPMs of chloramphenicol and erythromycin.

For overlap (Figures 2A,D), these antibiotic combinations are probably synergetic (87.5%, p−value=0.118 , permutation test). From the perspective of network pharmacology, if DAMPs of two drugs overlap, it indicates that the two drugs are pharmacologically similar (Cheng et al., 2019). For example, chloramphenicol and erythromycin both target the 50S ribosome, and their binding sites are the peptidyl transferase center (PTC) and the nascent peptide exit tunnel (NPET) on the 50S subunit, respectively (Lin et al., 2018). They can inhibit protein synthesis in a synergistic way (Figure 3B) (Chandrasekaran et al., 2016). As shown in Figure 3A, DAMPs of chloramphenicol and erythromycin have common nodes. Hence, the network proximity of the two DAMPs is negative, $S_{TET-CHL}=-0.97$ . To verify this idea, we performed virtual screening for nodes in the DAMP of trimethoprim (a dihydrofolate reductase inhibitor). Eventually, we identified a dihydropteroate synthase inhibitor—sulfamethoxazole. The DAMPs between sulfamethoxazole and trimethoprim overlap ( $S_{TRI-SUL}=-0.12$ , Supplementary Figure S2A). Previous studies have shown that a combination of trimethoprim and sulfamethoxazole not only interferes with folic acid synthesis synergistically (Yeh et al., 2006) but also reduces the risk of bacterial resistance (Pappas et al., 2009). In summary, synergistic drug combinations tend to act on the same biological pathways.

For separation (Figures 2B,E), these antibiotic combinations were almost not synergetic (90.1%, $p-value<10^{-4}$ , permutation test, see more from SI). In other words, the two drugs are pharmacologically distinct in this case. For example, nalidixic acid (an inhibitor of DNA gyrase) and chloramphenicol (an inhibitor of protein synthesis) take effect in different biological processes, so their DAMPs are topologically separated ( $S_{NAL-CHL}=0.92$ , Supplementary Figure S2B), and nalidixic acid and chloramphenicol do not show the synergy effect. Levofloxacin not only inhibits DNA gyrase but also inhibits DNA topoisomerase (Table 1). Hence, the DAMPs of levofloxacin and nalidixic acid overlap, resulting in the synergy effect. In addition, DAMPs of some synergistic drug combinations are topologically separated. This may result from the following reasons: a) experimental data itself: the correlation coefficient of the α-score between two replicates is only 0.81, which leads to a random error; b) some drugs have unknown targets: recent evidence suggests that gentamicin has a second binding site around H69 of the 23S rRNA of the 50S ribosome (Serio et al., 2018). This may be the reason for the synergy between gentamicin and tetracycline.

For identical (Figures 2C,F), these antibiotic combinations showed a definite additive effect (100%). For example, clarithromycin and erythromycin not only act on the same targets (Table 1) but also have similar chemical structures (98.1%, Tanimoto similarity; more details can be found in SI). Hence, we consider the two drugs to be pharmacologically identical, which leads to an additive effect.

To demonstrate the usefulness of the PPI network, an ablation test was performed where the PPI network was randomized. Supplementary Figure S3 shows that the randomized PPI network produces worse results, so an accurate PPI network is crucial for our model.

### Prediction of Synergistic Antibiotic Combinations by Graph Regularization

Graph regularization is a useful model to predict different relationships between various types of biological entities (Luo et al., 2018; Ding et al., 2020). Through the aforementioned analysis, we found that if two drugs are pharmacologically similar, then the drug pair is probably a synergistic antibiotic combination (Figure 2D). Therefore, we can define a loss function of *Y* (Eq. 8). However, if two drugs are pharmacologically identical (*S*_*AB*_ = −1), then the drug pair shows an additive effect (Figure 2C). Therefore, we set *W*_*ij*_ of these drug pairs to 0 (Eq. 9). Next, we used the aforementioned 14 antibiotics (Table 1) for the training set to predict interactions with the following three antibiotics (Table 2) by Eq. 12. The entire predicted scores are listed in Table 3. A larger predicted score of drug pairs suggests that they would probably be the synergistic antibiotic combinations. In Supplementary Tables S2–S6, we confirmed that the algorithm is not sensitive to the choice of γ , so it was simply fixed at 0.7. In Supplementary Figure S4, we demonstrated that the impact of the threshold changes on the performance of our method. When the threshold increases from 0.1 to 0.5, the precision increases, and the recall and accuracy decrease. When the threshold is larger than 0.2, the F1 decreases. Therefore, we set the threshold to be 0.2 in our model. Eight potential synergistic antibiotic combinations were found: TET-ROX, ROX-CLA, OXA-PNG, CEF-PNG, ROX-ERY, ROX-CHL, PNG-TET, and PNG-TRI. In the experiments conducted by Mason et al. (Mason et al., 2017), TET-ROX, ROX-CLA, OXA-PNG, CEF-PNG, and PNG-TET were identified as synergistic antibiotic combinations, and ROX-ERY, ROX-CHL, and PNG-TRI were additive. However, as alluded to above, there are random errors in experimental measurements, which might have some impact on the classification of antibiotic combinations. As expected, we found that ROX-ERY and ROX-CHL were identified as synergistic antibiotic combinations in the experiments by Yilancioglu (Yilancioglu, 2019). This means that our model has good stability for the prediction of synergistic antibiotic combinations.

**TABLE 2:** List of antibiotics used for the validation set and their targets and mechanisms.

Drug	Abbreviation	Targets	Mechanism of action
Kanamycin	KAN	rpsL Lin et al. (2018)	Protein synthesis, 30 S inhibition
Penicillin G	PNG	pbpG, dacB Kocaoglu and Carlson (2015)	Cell wall
Roxithromycin	ROX	rplD, rplV Salehi et al. (2020)	Protein synthesis, 50 S inhibition

**TABLE 3:** The entire predicted scores were calculated by a graph regularization model and synergistic antibiotic combinations are colored red.

Drug1	Drug2	Score	Drug1	Drug2	Score
KAN	AMK	0	PNG	CIP	0
KAN	GEN	0	PNG	LEV	0
KAN	TOB	0	PNG	NAL	0
KAN	TET	0	PNG	TRI	0.259
KAN	CLA	0	PNG	OXA	0.519 Mason et al. (2017)
KAN	ERY	0	PNG	CEF	0.519 Mason et al. (2017)
KAN	CHL	0	PNG	NIT	0
KAN	CIP	0	ROX	AMK	0.080
KAN	LEV	0	ROX	GEN	0.162
KAN	NAL	0	ROX	TOB	0
KAN	TRI	0	ROX	TET	0.485 Mason et al. (2017)
KAN	OXA	0 Mason et al. (2017)	ROX	CLA	0.405 Mason et al. (2017)
KAN	CEF	0	ROX	ERY	0.405 Yilancioglu (2019)
KAN	NIT	0	ROX	CHL	0.485 Yilancioglu (2019)
PNG	AMK	0	ROX	CIP	0
PNG	GEN	0	ROX	LEV	0
PNG	TOB	0	ROX	NAL	0
PNG	TET	0.259 Mason et al. (2017)	ROX	TRI	0
PNG	CLA	0	ROX	OXA	0.162
PNG	ERY	0 Mason et al. (2017)	ROX	CEF	0 Mason et al. (2017)
PNG	CHL	0	ROX	NIT	0

### Comparison With Other Methods

Previously, there have been studies to predict synergistic antibiotic combinations through computational methods. In this section, we compared the performance of our model (Eqs 13–16) with other methods, such as CosynE (Mason et al., 2017) and INDIGO (Chandrasekaran et al., 2016) on the benchmark dataset. As shown in Table 4, our model achieved better performance in terms of various metrics.

**TABLE 4:** Performance comparison of CosynE (Mason et al., 2017), INDIGO (Chandrasekaran et al., 2016), and our model.

	Precision	Recall	Accuracy	F1
CosynE	0.83	0.38	0.86	0.53
INDIGO	0.3	0.85	0.58	0.44
Our model	0.875	0.7	0.90	0.78

## Discussion

To reduce the cost and time of high-throughput drug combination experiments, we proposed a graph learning framework (Figure 1) to predict potential synergistic antibiotic combinations. First, we collected antibiotic combinations (Supplementary Table S1) and their corresponding targets (Table 1) from the literature. Network analysis revealed that the shortest distance between targets of antibiotic combinations was not sufficient to classify synergistic antibiotic combinations (Supplementary Figure S1). Therefore, we proposed a network proximity method combined with network propagation to quantify the relationships of antibiotic combinations (Figures 1B,C). An important finding is that synergistic antibiotic combinations have a specific network topological relationship, that is, the overlap pattern (Figure 2). This suggests that synergistic antibiotic combinations tend to act on the same biological pathways. Using the antibiotic combination erythromycin and chloramphenicol as a case study, we confirmed that the network proximity of their DAMPs is negative (Supplementary Table S1), and they can inhibit protein synthesis in a synergistic way (Figure 3B). In addition, the network proximity of each drug pair can be fed into the graph regularization model (Eq. 8) to predict new synergistic antibiotic combinations. Most of the predicted synergistic antibiotic combinations have been proven by a series of experiments (Table 3).

Previously, chemo-genomics data (Chandrasekaran et al., 2016) or structural compound fingerprints (Mason et al., 2017) have been used to build machine learning models and thereby predict antibiotic interactions between drug pairs. Based on the concepts proposed by these models, many potential synergistic antibiotic combinations were predicted and validated. However, the performance of these two methods is moderate (Table 4) because of the curse of dimensionality. Compared to these two approaches, our model is based on a feature at deeper molecular levels, the network proximity of DAMPs, which provides a more elegant and efficient way to describe the relationship of drug pairs. This not only makes our model have better predictability (Table 4) but also allows our model to achieve better interpretability. Even so, there are some limitations in our model. First, we focused on the paired antibiotic combinations. In the future, we will also investigate high-order drug combinations. Second, the PPI network is crucial for our model (Supplementary Figure S3). In the current model, an undirected network was used, and next, we will adopt a directed and signed network, which may be useful for improving the performance of our model.

## Conclusion

Antibiotic combinations provide a useful way to combat antibiotic resistance. In this study, we proposed a graph learning framework to understand the mechanisms of drug pairs and to predict synergistic antibiotic combinations. By quantifying the relationship between drug pairs based on the network proximity of DAMPs, a graph regularization model can identify potential synergistic antibiotic combinations. This allows us to explore the need for antibiotic combinations more effectively.

## Data Availability

The original contributions presented in the study are included in the article/Supplementary Material, further inquiries can be directed to the corresponding authors.

## References

[B1] Aracena P. Lazo-Hernández C. Molina-Berríos A. Sepúlveda D. R. Reinoso C. Larraín J. I. (2014). Microsomal Oxidative Stress Induced by NADPH Is Inhibited by Nitrofurantoin Redox Biotranformation. Free Radic. Res. 48 (2), 129–136. 10.3109/10715762.2013.836695 23967899

[B2] Author Anonymous (2013). The Antibiotic Alarm. Nature 495 (7440), 141. 10.1038/495141a 23495392

[B3] Buck M. A. Cooperman B. S. (1990). Single Protein Omission Reconstitution Studies of Tetracycline Binding to the 30S Subunit of *Escherichia coli* Ribosomes. Biochemistry 29 (22), 5374–5379. 10.1021/bi00474a024 2200507

[B4] Chandrasekaran S. Cokol-Cakmak M. Sahin N. Yilancioglu K. Kazan H. Collins J. J. (2016). Chemogenomics and Orthology-Based Design of Antibiotic Combination Therapies. Mol. Syst. Biol. 12 (5), 872. 10.15252/msb.20156777 27222539 PMC5289223

[B5] Cheng F. Kovács I. A. Barabási A. L. (2019). Network-based Prediction of Drug Combinations. Nat. Commun. 10, 1197. 10.1038/s41467-019-09186-x 30867426 PMC6416394

[B6] Cokol M. Chua H. N. Tasan M. Mutlu B. Weinstein Z. B. Suzuki Y. (2011). Systematic Exploration of Synergistic Drug Pairs. Mol. Syst. Bio 7 (1), 544. 10.1038/msb.2011.71 22068327 PMC3261710

[B7] Cokol M. Li C. Chandrasekaran S. (2018). Chemogenomic Model Identifies Synergistic Drug Combinations Robust to the Pathogen Microenvironment. PLOS COMPUTATIONAL BIOLOGY 14 (12), e1006677. 10.1371/journal.pcbi.1006677 30596642 PMC6329523

[B8] Csermely P. Korcsmáros T. Kiss H. J. London G. Nussinov R. (2013). Structure and Dynamics of Molecular Networks: A Novel Paradigm of Drug Discovery: A Comprehensive Review. Pharmacol. Ther. 138 (3), 333–408. 10.1016/j.pharmthera.2013.01.016 23384594 PMC3647006

[B9] Ding P. Shen C. Lai Z. Liang C. Li G. Luo J. (2020). Incorporating Multisource Knowledge to Predict Drug Synergy Based on Graph Co-regularization. J. Chem. Inf. Model. 60 (1), 37–46. 10.1021/acs.jcim.9b00793 31891264

[B10] Farha M. A. French S. Brown E. D. (2021). Systems-Level Chemical Biology to Accelerate Antibiotic Drug Discovery. Acc. Chem. Res. 54 (8), 1909–1920. 10.1021/acs.accounts.1c00011 33787225

[B11] Hopkins A. L. (2008). Network Pharmacology: the Next Paradigm in Drug Discovery. Nat. Chem. Biol. 4 (11), 682–690. 10.1038/nchembio.118 18936753

[B12] Kocaoglu O. Carlson E. E. (2015). Profiling of β-lactam Selectivity for Penicillin-Binding Proteins in *Escherichia coli* Strain DC2. Antimicrob. Agents Chemother. 59 (5), 2785–2790. 10.1128/AAC.04552-14 25733506 PMC4394777

[B13] Li S. Zhang F. Xiao X. Guo Y. Wen Z. Li M. (2021). Prediction of Synergistic Drug Combinations for Prostate Cancer by Transcriptomic and Network Characteristics. Front. Pharmacol. 12 (315), 634097. 10.3389/fphar.2021.634097 33986671 PMC8112211

[B14] Lin J. Zhou D. Steitz T. A. Polikanov Y. S. Gagnon M. G. (2018). Ribosome-Targeting Antibiotics: Modes of Action, Mechanisms of Resistance, and Implications for Drug Design. Annu. Rev. Biochem. 87 (1), 451–478. 10.1146/annurev-biochem-062917-011942 29570352 PMC9176271

[B15] Luo J. Ding P. Liang C. Chen X. (2018). Semi-supervised Prediction of Human miRNA-Disease Association Based on Graph Regularization Framework in Heterogeneous Networks. Neurocomputing 294, 29–38. 10.1016/j.neucom.2018.03.003

[B16] Lv J. Deng S. Zhang L. (2021). A Review of Artificial Intelligence Applications for Antimicrobial Resistance. Biosafety and Health 3 (1), 22–31. 10.1016/j.bsheal.2020.08.003

[B17] Maan M. K. Chaudhry T. H. Sattar A. Shabbir M. A. B. Ahmed S. Mi K. (2021). Dose Optimization of Aditoprim-Sulfamethoxazole Combinations against Trueperella Pyogenes from Patients with Clinical Endometritis by Using Semi-mechanistic PK/PD Model. Front. Pharmacol. 12 (3099), 753359. 10.3389/fphar.2021.753359 34867364 PMC8635024

[B18] Mason D. J. Stott I. Ashenden S. Weinstein Z. B. Karakoc I. Meral S. (2017). Prediction of Antibiotic Interactions Using Descriptors Derived from Molecular Structure. J. Med. Chem. 60 (9), 3902–3912. 10.1021/acs.jmedchem.7b00204 28383902

[B19] Mason D. J. Eastman R. T. Lewis R. P. I. Stott I. P. Guha R. Bender A. (2018). Using Machine Learning to Predict Synergistic Antimalarial Compound Combinations with Novel Structures. Front. Pharmacol. 9, 1096. 10.3389/fphar.2018.01096 30333748 PMC6176478

[B20] Mathers A. J. (2015). Antibiotics in Laboratory Medicine, 6th Edition. Clin. Infect. Dis. 60 (9), 1446–1447. 10.1093/cid/civ078

[B21] Menche J. Sharma A. Kitsak M. Ghiassian S. D. Vidal M. Loscalzo J. (2015). Disease Networks. Uncovering Disease-Disease Relationships through the Incomplete Interactome. Science 347 (6224), 1257601. 10.1126/science.1257601 25700523 PMC4435741

[B22] Mi H. Muruganujan A. Ebert D. Huang X. Thomas P. D. (2018). PANTHER Version 14: More Genomes, a New PANTHER GO-Slim and Improvements in Enrichment Analysis Tools. Nucleic Acids Res. 47 (D1), D419–D426. 10.1093/nar/gky1038 PMC6323939 30407594

[B23] Murray C. J. L. Ikuta K. S. Sharara F. Swetschinski L. Robles Aguilar G. Gray A. (2022). Global burden of Bacterial Antimicrobial Resistance in 2019: a Systematic Analysis. The Lancet 399, 629–655. 10.1016/S0140-6736(21)02724-0 PMC8841637 35065702

[B24] Nichols R. J. Sen S. Choo Y. J. Beltrao P. Zietek M. Chaba R. (2011). Phenotypic Landscape of a Bacterial Cell. Cell 144 (1), 143–156. 10.1016/j.cell.2010.11.052 21185072 PMC3060659

[B25] Odds F. C. (2003). Synergy, Antagonism, and what the Chequerboard Puts between Them. J. Antimicrob. Chemother. 52 (1), 1. 10.1093/jac/dkg301 12805255

[B26] Onodera Y. Okuda J. Tanaka M. Sato K. (2002). Inhibitory Activities of Quinolones against DNA Gyrase and Topoisomerase IV of *Enterococcus faecalis* . Antimicrob. Agents Chemother. 46 (6), 1800–1804. 10.1128/AAC.46.6.1800-1804.2002 12019093 PMC127212

[B27] Pan X. S. Ambler J. Mehtar S. Fisher L. M. (1996). Involvement of Topoisomerase IV and DNA Gyrase as Ciprofloxacin Targets in Streptococcus Pneumoniae. Antimicrob. Agents Chemother. 40 (10), 2321–2326. 10.1128/AAC.40.10.2321 8891138 PMC163528

[B28] Pappas G. Athanasoulia A. P. Matthaiou D. K. Falagas M. E. (2009). Trimethoprim-sulfamethoxazole for Methicillin-Resistant *Staphylococcus aureus*: a Forgotten Alternative? J. Chemother. 21 (2), 115–126. 10.1179/joc.2009.21.2.115 19423463

[B29] Pongs O. Bald R. Erdmann V. A. (1973). Identification of Chloramphenicol-Binding Protein in *Escherichia coli* Ribosomes by Affinity Labeling. Proc. Natl. Acad. Sci. U S A. 70 (8), 2229–2233. 10.1073/pnas.70.8.2229 4599619 PMC433707

[B30] Salehi N. Attaran B. Zare-Mirakabad F. Ghadiri B. Esmaeili M. Shakaram M. (2020). The Outward Shift of Clarithromycin Binding to the Ribosome in Mutant *Helicobacter pylori* Strains. Helicobacter 25 (6), e12731. 10.1111/hel.12731 32794288

[B31] Saraswathi V. Amit G. Priti H. (2009). Intra and Inter-molecular Communications through Protein Structure Network. Curr. Protein Pept. Sci. 10 (2), 146–160. 10.2174/138920309787847590 19355982

[B32] Serio A. W. Keepers T. Andrews L. Krause K. M. Bush K. (2018). Aminoglycoside Revival: Review of a Historically Important Class of Antimicrobials Undergoing Rejuvenation. EcoSal Plus 8 (1). 10.1128/ecosalplus.ESP-0002-2018 PMC11575671 30447062

[B33] Shen L. L. Pernet A. G. (1985). Mechanism of Inhibition of DNA Gyrase by Analogues of Nalidixic Acid: the Target of the Drugs Is DNA. Proc. Natl. Acad. Sci. U S A. 82 (2), 307–311. 10.1073/pnas.82.2.307 2982149 PMC397026

[B34] Sheng Z. Sun Y. Yin Z. Tang K. Cao Z. (2017). Advances in Computational Approaches in Identifying Synergistic Drug Combinations. Brief Bioinform 19 (6), 1172–1182. 10.1093/bib/bbx047 28475767

[B35] Shin D. Lee J. Gong J. R. Cho K. H. (2017). Percolation Transition of Cooperative Mutational Effects in Colorectal Tumorigenesis. Nat. Commun. 8 (1), 1270. 10.1038/s41467-017-01171-6 29097710 PMC5668266

[B36] Szklarczyk D. Gable A. L. Nastou K. C. Lyon D. Kirsch R. Pyysalo S. (2020). The STRING Database in 2021: Customizable Protein-Protein Networks, and Functional Characterization of User-Uploaded Gene/measurement Sets. Nucleic Acids Res. 49 (D1), D605–D612. 10.1093/nar/gkaa1074 PMC7779004 33237311

[B37] Tyers M. Wright G. D. (2019). Drug Combinations: a Strategy to Extend the Life of Antibiotics in the 21st century. Nat. Rev. Microbiol. 17 (3), 141–155. 10.1038/s41579-018-0141-x 30683887

[B38] Vanunu O. Magger O. Ruppin E. Shlomi T. Sharan R. (2010). Associating Genes and Protein Complexes with Disease via Network Propagation. Plos Comput. Biol. 6 (1), e1000641. 10.1371/journal.pcbi.1000641 20090828 PMC2797085

[B39] Ventola C. L. (2015). The Antibiotic Resistance Crisis: Part 1: Causes and Threats. P T 40 (4), 277–283. 10.1016/S0194-5998(97)80284-7 25859123 PMC4378521

[B40] Wang J. Luo L. Ding Q. Wu Z. Peng Y. Li J. (2021a). Development of a Multi-Target Strategy for the Treatment of Vitiligo via Machine Learning and Network Analysis Methods. Front. Pharmacol. 12 (2524), 754175. 10.3389/fphar.2021.754175 34603063 PMC8479195

[B41] Wang Y. Yang H. Chen L. Jafari M. Tang J. (2021b). Network-based Modeling of Herb Combinations in Traditional Chinese Medicine. Brief. Bioinf. 22 (5), bbab106. 10.1093/bib/bbab106 PMC8425426 33834186

[B42] Weinstein Z. B. Bender A. Cokol M. (2017). Prediction of Synergistic Drug Combinations. Curr. Opin. Syst. Biol. 4, 24–28. 10.1016/j.coisb.2017.05.005

[B43] Wekselman I. Zimmerman E. Davidovich C. Belousoff M. Matzov D. Krupkin M. (2017). The Ribosomal Protein uL22 Modulates the Shape of the Protein Exit Tunnel. Structure 25 (8), 1233–e3. 10.1016/j.str.2017.06.004 28689968

[B44] Wishart D. S. Feunang Y. D. Guo A. C. Lo E. J. Marcu A. Grant J. R. (2017). DrugBank 5.0: a Major Update to the DrugBank Database for 2018. Nucleic Acids Res. 46 (D1), D1074–D1082. 10.1093/nar/gkx1037 PMC5753335 29126136

[B45] Wright G. D. (2014). Something Old, Something New: Revisiting Natural Products in Antibiotic Drug Discovery. Can. J. Microbiol. 60 (3), 147–154. 10.1139/cjm-2014-0063 24588388

[B46] Wróbel A. Arciszewska K. Maliszewski D. Drozdowska D. (2020). Trimethoprim and Other Nonclassical Antifolates an Excellent Template for Searching Modifications of Dihydrofolate Reductase Enzyme Inhibitors. J. Antibiot. (Tokyo) 73 (1), 5–27. 10.1038/s41429-019-0240-6 31578455 PMC7102388

[B47] Yeh P. Tschumi A. I. Kishony R. (2006). Functional Classification of Drugs by Properties of Their Pairwise Interactions. Nat. Genet. 38 (4), 489–494. 10.1038/ng1755 16550172

[B48] Yilancioglu K. (2019). Antimicrobial Drug Interactions: Systematic Evaluation of Protein and Nucleic Acid Synthesis Inhibitors. Antibiotics (Basel) 8 (3), 114. 10.3390/antibiotics8030114 PMC6784067 31405069

[B49] Zhabiz G. Omar B. Donald Gene P. (2014). Bacteriophage Therapy: a Potential Solution for the Antibiotic Resistance Crisis. The J. Infect. Developing Countries 8 (02), 129–136. 10.3855/jidc.3573 24518621

[B50] Zhang L. Lv J. Xiao M. Yang L. Zhang L. (2021). Exploring the Underlying Mechanism of Action of a Traditional Chinese Medicine Formula, Youdujing Ointment, for Cervical Cancer Treatment. Quant. Biol. 9 (3), 292–303. 10.15302/j-qb-021-0236

