# Supplementary material for: Prediction of Synergistic Antibiotic Combinations by Graph Learning
Source: Front Pharmacol. 2022 Mar 8;13:849006. doi: 10.3389/fphar.2022.849006 (PMC8958015; doi:10.3389/fphar.2022.849006)
Supplement: Supplementary file 1 [file DataSheet1.docx]

Supplementary Material

# Supplementary Data

## Molecular structure similarity

We used Tanimoto coefficient (*T*, Eq. 1) to evaluate chemical structure similarity of drug combinations.

| $T=\frac{c}{a+b-c}$ | (1) |
| --- | --- |

where a and b are molecular fingerprints of drug A and drug B, respectively. *c* is the same elements of *a* and *b*. Molecular fingerprints of drugs were calculated by RDKit ([Landrum, 2019](#_ENREF_1)), representing each drug as 166 MACCS keys.

## Permutation tests

Permutation test is a method of statistical inference using random sample ([Oden and Wedel, 1975](#_ENREF_2)). This method has good performance even if sample sizes are few. For the second column in Figure 2, we divide drug combinations into two groups, synergy or not synergy. And then performed 1,000 randomizations.

## The detail of solving the loss function

$$E\left( Y \right)=\frac{1}{2}\left( \sum_{i,j=1}^{l+u} W_{ij}||\frac{Y_{i}}{\sqrt{d_{i}}}-\frac{Y_{j}}{\sqrt{d_{j}}}||^{2} \right)+\mu\sum_{i=1}^{l} ||Y_{i}-\hat{Y}_{i}||^{2}$$

$$=\frac{1}{2}\sum_{i,j=1}^{l+u} W_{ij}(\frac{Y_{i}^{2}}{d_{i}}-\frac{2Y_{i}Y_{j}}{\sqrt{d_{i}d_{j}}}+\frac{Y_{j}^{2}}{d_{j}})+\mu\sum_{i=1}^{l} ||Y_{i}-\hat{Y}_{i}||^{2}$$

$$=\frac{1}{2}[\sum_{i=1}^{l+u} Y_{i}^{2}-\sum_{i,j=1}^{l+u} \frac{2{W_{ij}Y}_{i}Y_{j}}{\sqrt{d_{i}d_{j}}}+\sum_{j=1}^{l+u} Y_{j}^{2}]+\mu\sum_{i=1}^{l} ||Y_{i}-\hat{Y}_{i}||^{2}$$

$$=Y^{2}-QY^{2}+\mu(Y-\hat{Y})^{2}$$

Thus,

$$\left. \frac{\partial E(Y)}{\partial Y} \right|_{Y=Y^{*}}=Y^{*}-QY^{*}+\mu(Y^{*}-\hat{Y})=0$$

Which can be transformed into

$$Y^{*}-\frac{1}{1+\mu}QY^{*}-\frac{\mu}{1+\mu}\hat{Y}=0$$

Thus,

$$\left( I-\alpha Q \right)Y^{*}=\beta\hat{Y}$$

Where $\alpha=\frac{1}{1+\mu}$ and $\beta=\frac{\mu}{1+\mu}$. Since $I-\alpha Q$ is invertible, we have

$$Y^{*}=\beta(I-\alpha Q)^{-1}\hat{Y}$$

## The proof that $\boldsymbol{I}\mathbf{-}\boldsymbol{\alpha Q}$ is positive definite matrix and invertible

$$I\boldsymbol{-}\alpha Q=\alpha(\frac{1-\alpha}{\alpha}I+I-Q)$$

Since $\alpha\epsilon[0,1]$ we just need to proof that $I-Q$ is positive semi-definite matrix

$$I-Q=I-D^{-\frac{1}{2}}WD^{-\frac{1}{2}}$$

$$=D^{-\frac{1}{2}}\left( D-W \right)D^{-\frac{1}{2}}$$

$$=D^{-\frac{1}{2}}LD^{-\frac{1}{2}}$$

$$f^{T}Lf=f^{T}Df-f^{T}Wf=\sum_{i=1}^{n} d_{i}f_{i}^{2}-\sum_{i,j=1}^{n} w_{ij}f_{i}f_{j}$$

$$=\frac{1}{2}\left( \sum_{i=1}^{n} d_{i}f_{i}^{2}-2\sum_{i,j=1}^{n} w_{ij}f_{i}f_{j}+\sum_{j=1}^{n} d_{j}f_{j}^{2} \right)$$

$$=\frac{1}{2}\sum_{i,j=1}^{n} w_{ij}(f_{i}-f_{j})^{2}\geq0$$

# Supplementary Figures and Tables

## Supplementary Figures


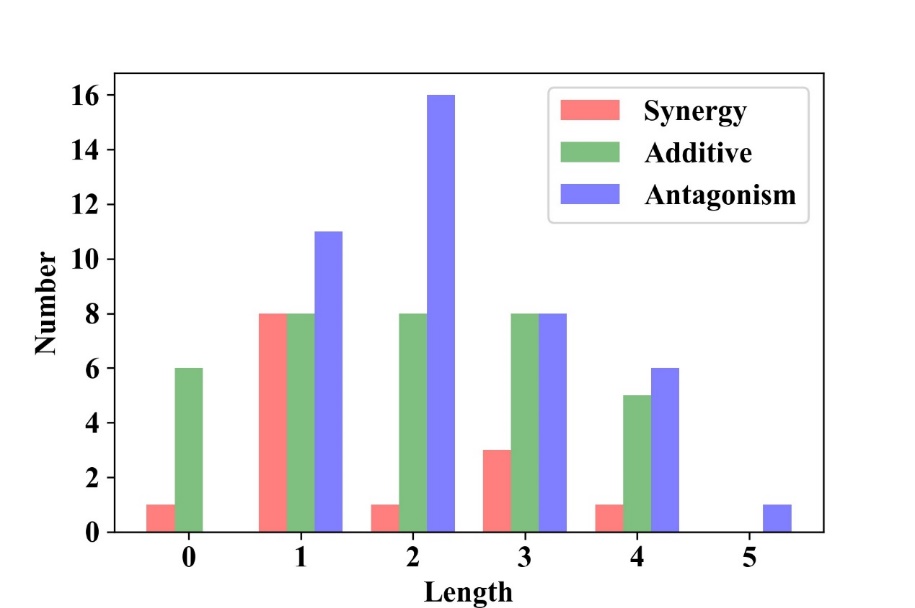


**Figure S1**. The number of synergistic, additive, and antagonistic drug combinations for corresponding length in the PPI network.


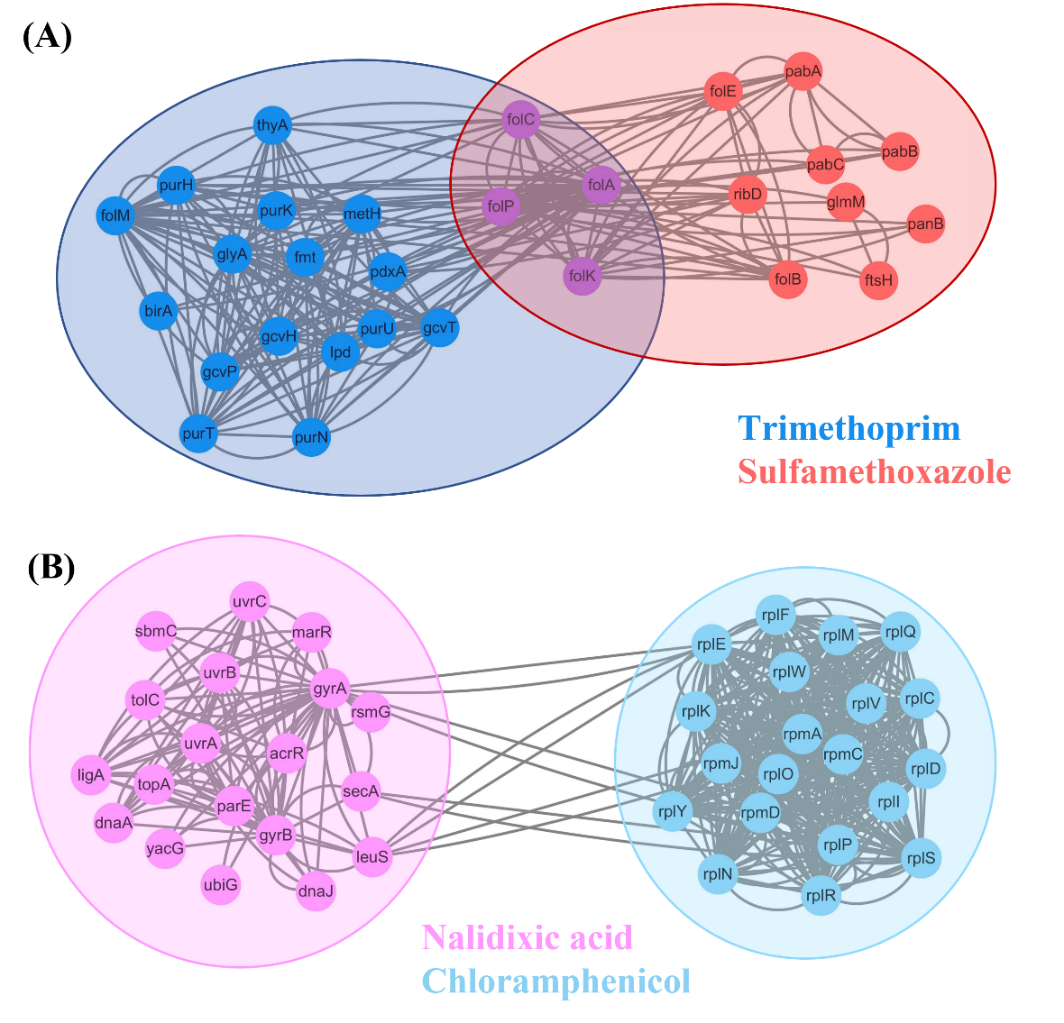


**Figure S2**. Subnetworks of the network-based relationship between DAPMs of (A) trimethoprim and sulfamethoxazole, and (B) nalidixic acid and chloramphenicol.


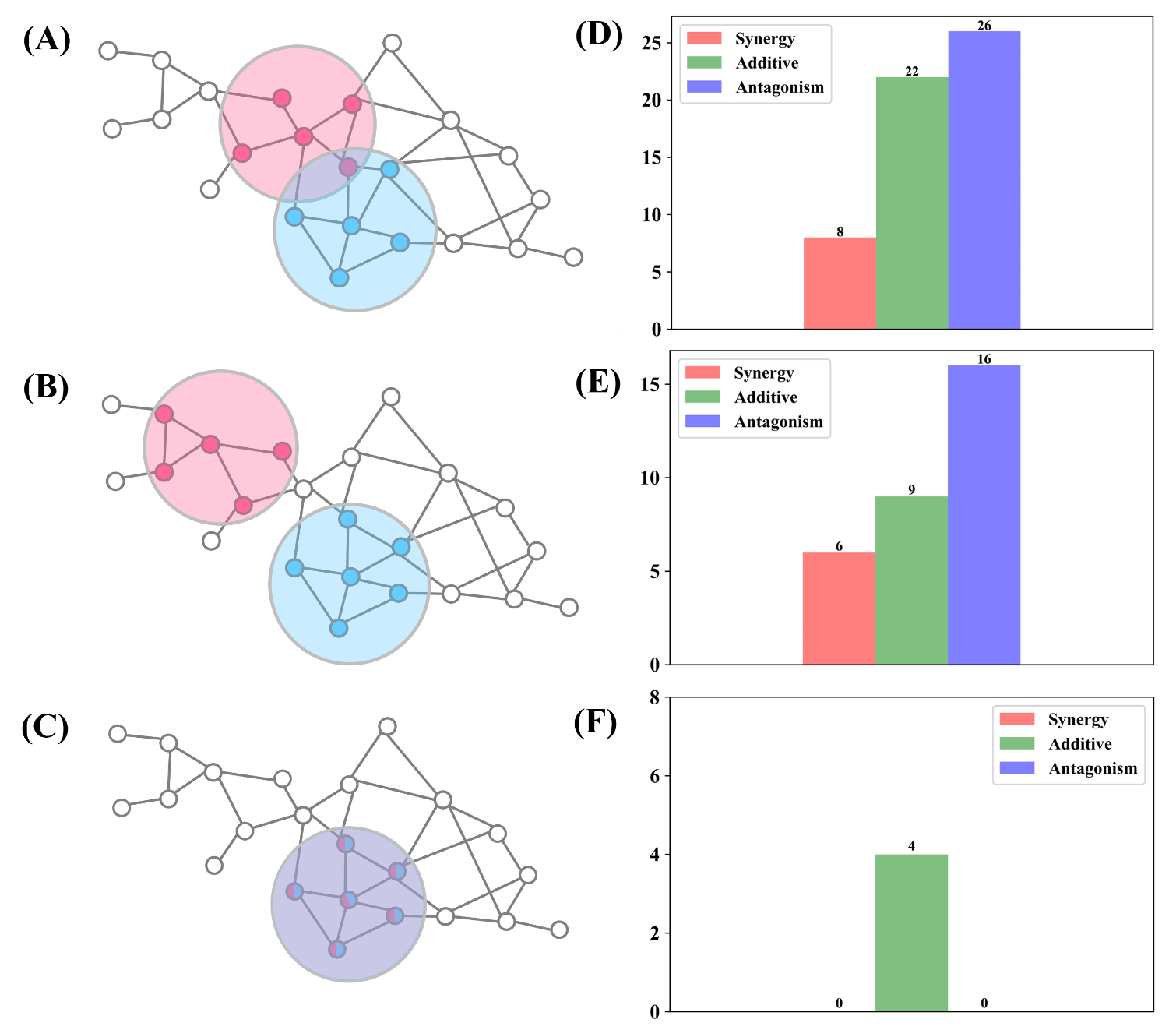


**Figure S3.** The relationships between drug interaction and network structures in randomized PPI network. (A, B, C) Sketch map of the three topologically distinct classes. (D, E, F) the number of synergistic, additive, and antagonistic drug combinations for corresponding network structure.

**Figure S4.** Performance of our method in different thresholds

## Supplementary Table

**Table S1.** α-score, type, length of the shortest pathway, SAB, and JAB of all 91 drug pairs used for network analysis.

| **Drug1** | **Drug2** | **α-score** | **Type** | **Length** | **S_AB_** | **J_AB_** | **T_AB_** |
| --- | --- | --- | --- | --- | --- | --- | --- |
| AMK | TOB | 0.53 | Additive | 0.00 | -1.00 | 1.00 | 0.87 |
| AMK | GEN | 0.73 | Additive | 0.00 | -1.00 | 1.00 | 0.72 |
| AMK | ERY | 1.43 | Antagonism | 1.00 | 0.95 | 0.00 | 0.53 |
| AMK | CLA | -0.33 | Synergy | 1.00 | 0.95 | 0.00 | 0.51 |
| AMK | CIP | -0.02 | Additive | 1.00 | 0.95 | 0.00 | 0.48 |
| AMK | LEV | 0.66 | Additive | 1.00 | 0.95 | 0.00 | 0.44 |
| AMK | TET | 0.78 | Additive | 1.00 | 0.96 | 0.00 | 0.43 |
| AMK | NAL | 0.71 | Additive | 1.00 | 0.96 | 0.00 | 0.38 |
| AMK | CHL | 2.73 | Antagonism | 1.00 | 0.96 | 0.00 | 0.44 |
| AMK | TRI | -0.34 | Synergy | 2.00 | 0.96 | 0.00 | 0.44 |
| AMK | CEF | 1.37 | Antagonism | 2.00 | 1.95 | 0.00 | 0.55 |
| AMK | OXA | 0.54 | Additive | 3.00 | 1.10 | 0.00 | 0.38 |
| AMK | NIT | -0.42 | Synergy | 4.00 | 2.24 | 0.00 | 0.43 |
| CEF | NIT | 2.89 | Antagonism | 4.00 | 2.78 | 0.00 | 0.44 |
| CHL | CIP | 2.25 | Antagonism | 2.00 | 0.91 | 0.00 | 0.34 |
| CHL | LEV | 4.39 | Antagonism | 2.00 | 0.91 | 0.00 | 0.31 |
| CHL | NAL | 2.71 | Antagonism | 2.00 | 0.92 | 0.00 | 0.27 |
| CHL | CEF | 0.77 | Additive | 2.00 | 1.00 | 0.00 | 0.37 |
| CHL | OXA | 1.21 | Antagonism | 3.00 | 1.00 | 0.00 | 0.37 |
| CHL | TRI | 2.70 | Antagonism | 3.00 | 1.00 | 0.00 | 0.27 |
| CHL | NIT | 1.25 | Antagonism | 4.00 | 2.00 | 0.00 | 0.54 |
| CIP | LEV | -0.01 | Additive | 0.00 | -1.00 | 1.00 | 0.78 |
| CIP | NAL | 0.24 | Additive | 0.00 | -0.24 | 0.38 | 0.58 |
| CIP | TRI | -0.47 | Synergy | 3.00 | 0.47 | 0.00 | 0.41 |
| CIP | CEF | 1.06 | Antagonism | 2.00 | 0.44 | 0.00 | 0.44 |
| CIP | OXA | 0.77 | Additive | 3.00 | 0.16 | 0.00 | 0.44 |
| CIP | NIT | 1.71 | Antagonism | 4.00 | 1.91 | 0.00 | 0.37 |
| CLA | ERY | -0.17 | Additive | 0.00 | -1.00 | 1.00 | 0.98 |
| CLA | CHL | -0.72 | Synergy | 1.00 | -0.97 | 0.57 | 0.31 |
| CLA | LEV | 1.39 | Antagonism | 2.00 | 0.93 | 0.00 | 0.49 |
| CLA | CIP | 2.15 | Antagonism | 2.00 | 0.93 | 0.00 | 0.40 |
| CLA | NAL | 1.11 | Antagonism | 2.00 | 0.94 | 0.00 | 0.48 |
| CLA | CEF | 0.17 | Additive | 2.00 | 1.00 | 0.00 | 0.50 |
| CLA | OXA | 0.77 | Additive | 3.00 | 1.00 | 0.00 | 0.44 |
| CLA | TRI | 2.89 | Antagonism | 3.00 | 1.00 | 0.00 | 0.41 |
| CLA | NIT | 0.19 | Additive | 4.00 | 2.00 | 0.00 | 0.32 |
| ERY | CHL | -0.74 | Synergy | 1.00 | -0.97 | 0.57 | 0.32 |
| ERY | LEV | 0.90 | Additive | 2.00 | 0.93 | 0.00 | 0.49 |
| ERY | CIP | 1.40 | Antagonism | 2.00 | 0.93 | 0.00 | 0.41 |
| ERY | NAL | 1.82 | Antagonism | 2.00 | 0.94 | 0.00 | 0.47 |
| ERY | CEF | 0.10 | Additive | 2.00 | 1.00 | 0.00 | 0.51 |
| ERY | OXA | 1.13 | Antagonism | 3.00 | 1.00 | 0.00 | 0.44 |
| ERY | TRI | 0.87 | Additive | 3.00 | 1.00 | 0.00 | 0.41 |
| ERY | NIT | 0.59 | Additive | 4.00 | 2.00 | 0.00 | 0.33 |
| GEN | TOB | -0.13 | Additive | 0.00 | -1.00 | 1.00 | 0.72 |
| GEN | ERY | 0.42 | Additive | 1.00 | 0.95 | 0.00 | 0.69 |
| GEN | CLA | 0.44 | Additive | 1.00 | 0.95 | 0.00 | 0.68 |
| GEN | LEV | 0.09 | Additive | 1.00 | 0.95 | 0.00 | 0.47 |
| GEN | CIP | 1.15 | Antagonism | 1.00 | 0.95 | 0.00 | 0.40 |
| GEN | TET | -0.66 | Synergy | 1.00 | 0.96 | 0.00 | 0.50 |
| GEN | NAL | 1.47 | Antagonism | 1.00 | 0.96 | 0.00 | 0.35 |
| GEN | TRI | 0.60 | Additive | 2.00 | 0.96 | 0.00 | 0.50 |
| GEN | CHL | 1.94 | Antagonism | 1.00 | 0.96 | 0.00 | 0.32 |
| GEN | CEF | 0.30 | Additive | 2.00 | 1.94 | 0.00 | 0.48 |
| GEN | OXA | 0.04 | Additive | 3.00 | 1.10 | 0.00 | 0.39 |
| GEN | NIT | 0.02 | Additive | 4.00 | 2.24 | 0.00 | 0.34 |
| LEV | NAL | -0.78 | Synergy | 0.00 | -0.24 | 0.38 | 0.60 |
| LEV | TRI | -0.09 | Additive | 3.00 | 0.47 | 0.00 | 0.41 |
| LEV | CEF | 3.05 | Antagonism | 2.00 | 0.44 | 0.00 | 0.44 |
| LEV | OXA | 1.36 | Antagonism | 3.00 | 0.16 | 0.00 | 0.47 |
| LEV | NIT | 1.18 | Antagonism | 4.00 | 1.91 | 0.00 | 0.37 |
| NAL | OXA | 1.48 | Antagonism | 3.00 | 0.85 | 0.00 | 0.45 |
| NAL | CEF | 2.03 | Antagonism | 2.00 | 1.69 | 0.00 | 0.42 |
| NAL | TRI | 1.21 | Antagonism | 3.00 | 1.05 | 0.00 | 0.52 |
| NAL | NIT | 1.96 | Antagonism | 4.00 | 1.96 | 0.00 | 0.34 |
| OXA | CEF | -0.33 | Synergy | 1.00 | -0.43 | 0.36 | 0.57 |
| OXA | NIT | 3.35 | Antagonism | 5.00 | 2.72 | 0.00 | 0.46 |
| TET | CLA | -0.91 | Synergy | 1.00 | -0.06 | 0.07 | 0.59 |
| TET | ERY | -0.79 | Synergy | 1.00 | -0.06 | 0.07 | 0.58 |
| TET | CHL | -0.68 | Synergy | 1.00 | -0.05 | 0.05 | 0.40 |
| TET | LEV | 2.73 | Antagonism | 2.00 | 0.76 | 0.00 | 0.44 |
| TET | CIP | 4.26 | Antagonism | 2.00 | 0.76 | 0.00 | 0.40 |
| TET | NAL | 3.16 | Antagonism | 2.00 | 0.89 | 0.00 | 0.41 |
| TET | TRI | 2.17 | Antagonism | 3.00 | 1.00 | 0.00 | 0.41 |
| TET | OXA | -0.79 | Synergy | 3.00 | 1.03 | 0.00 | 0.48 |
| TET | CEF | -0.03 | Additive | 2.00 | 1.04 | 0.00 | 0.51 |
| TET | NIT | 1.61 | Antagonism | 4.00 | 2.36 | 0.00 | 0.31 |
| TOB | ERY | 1.50 | Antagonism | 1.00 | 0.95 | 0.00 | 0.55 |
| TOB | CLA | 0.94 | Additive | 1.00 | 0.95 | 0.00 | 0.53 |
| TOB | CIP | 2.91 | Antagonism | 1.00 | 0.95 | 0.00 | 0.47 |
| TOB | LEV | 2.42 | Antagonism | 1.00 | 0.95 | 0.00 | 0.43 |
| TOB | NAL | 2.87 | Antagonism | 1.00 | 0.96 | 0.00 | 0.38 |
| TOB | TET | 1.24 | Antagonism | 1.00 | 0.96 | 0.00 | 0.38 |
| TOB | TRI | 0.36 | Additive | 2.00 | 0.96 | 0.00 | 0.48 |
| TOB | CHL | 3.38 | Antagonism | 1.00 | 0.96 | 0.00 | 0.39 |
| TOB | CEF | 1.51 | Antagonism | 2.00 | 1.94 | 0.00 | 0.50 |
| TOB | OXA | 0.60 | Additive | 3.00 | 1.10 | 0.00 | 0.33 |
| TOB | NIT | 0.15 | Additive | 4.00 | 2.24 | 0.00 | 0.38 |
| TRI | CEF | 0.19 | Additive | 3.00 | 1.37 | 0.00 | 0.38 |
| TRI | OXA | -1.33 | Synergy | 3.00 | 1.10 | 0.00 | 0.38 |
| TRI | NIT | -0.20 | Additive | 4.00 | 1.59 | 0.00 | 0.32 |

**Table S2.** The entire predicted scores ($\gamma=0.3$) were calculated by graph regularization model and synergistic antibiotic combinations were colored red.

| **Drug1** | **Drug2** | **score** | **Drug1** | **Drug2** | **score** |
| --- | --- | --- | --- | --- | --- |
| KAN | AMK | 0 | PNG | CIP | 0 |
| KAN | GEN | 0 | PNG | LEV | 0 |
| KAN | TOB | 0 | PNG | NAL | 0 |
| KAN | TET | 0 | PNG | TRI | 0.13043 |
| KAN | CLA | 0 | PNG | OXA | 0.26087 |
| KAN | ERY | 0 | PNG | CEF | 0.26087 |
| KAN | CHL | 0 | PNG | NIT | 0 |
| KAN | CIP | 0 | ROX | AMK | 0.01837 |
| KAN | LEV | 0 | ROX | GEN | 0.08658 |
| KAN | NAL | 0 | ROX | TOB | 0 |
| KAN | TRI | 0 | ROX | TET | 0.2099 |
| KAN | OXA | 0 | ROX | CLA | 0.19154 |
| KAN | CEF | 0 | ROX | ERY | 0.19154 |
| KAN | NIT | 0 | ROX | CHL | 0.2099 |
| PNG | AMK | 0 | ROX | CIP | 0 |
| PNG | GEN | 0 | ROX | LEV | 0 |
| PNG | TOB | 0 | ROX | NAL | 0 |
| PNG | TET | 0.13043 | ROX | TRI | 0 |
| PNG | CLA | 0 | ROX | OXA | 0.08658 |
| PNG | ERY | 0 | ROX | CEF | 0 |
| PNG | CHL | 0 | ROX | NIT | 0 |

**Table S3.** The entire predicted scores ($\gamma=0.4$) were calculated by graph regularization model and synergistic antibiotic combinations were colored red.

| **Drug1** | **Drug2** | **score** | **Drug1** | **Drug2** | **score** |
| --- | --- | --- | --- | --- | --- |
| KAN | AMK | 0 | PNG | CIP | 0 |
| KAN | GEN | 0 | PNG | LEV | 0 |
| KAN | TOB | 0 | PNG | NAL | 0 |
| KAN | TET | 0 | PNG | TRI | 0.16667 |
| KAN | CLA | 0 | PNG | OXA | 0.33333 |
| KAN | ERY | 0 | PNG | CEF | 0.33333 |
| KAN | CHL | 0 | PNG | NIT | 0 |
| KAN | CIP | 0 | ROX | AMK | 0.03077 |
| KAN | LEV | 0 | ROX | GEN | 0.10879 |
| KAN | NAL | 0 | ROX | TOB | 0 |
| KAN | TRI | 0 | ROX | TET | 0.27911 |
| KAN | OXA | 0 | ROX | CLA | 0.24834 |
| KAN | CEF | 0 | ROX | ERY | 0.24834 |
| KAN | NIT | 0 | ROX | CHL | 0.27911 |
| PNG | AMK | 0 | ROX | CIP | 0 |
| PNG | GEN | 0 | ROX | LEV | 0 |
| PNG | TOB | 0 | ROX | NAL | 0 |
| PNG | TET | 0.16667 | ROX | TRI | 0 |
| PNG | CLA | 0 | ROX | OXA | 0.10879 |
| PNG | ERY | 0 | ROX | CEF | 0 |
| PNG | CHL | 0 | ROX | NIT | 0 |

**Table S4.** The entire predicted scores ($\gamma=0.5$) were calculated by graph regularization model and synergistic antibiotic combinations were colored red.

| **Drug1** | **Drug2** | **score** | **Drug1** | **Drug2** | **score** |
| --- | --- | --- | --- | --- | --- |
| KAN | AMK | 0 | PNG | CIP | 0 |
| KAN | GEN | 0 | PNG | LEV | 0 |
| KAN | TOB | 0 | PNG | NAL | 0 |
| KAN | TET | 0 | PNG | TRI | 0.2 |
| KAN | CLA | 0 | PNG | OXA | 0.4 |
| KAN | ERY | 0 | PNG | CEF | 0.4 |
| KAN | CHL | 0 | PNG | NIT | 0 |
| KAN | CIP | 0 | ROX | AMK | 0.04545 |
| KAN | LEV | 0 | ROX | GEN | 0.12856 |
| KAN | NAL | 0 | ROX | TOB | 0 |
| KAN | TRI | 0 | ROX | TET | 0.34804 |
| KAN | OXA | 0 | ROX | CLA | 0.30258 |
| KAN | CEF | 0 | ROX | ERY | 0.30258 |
| KAN | NIT | 0 | ROX | CHL | 0.34804 |
| PNG | AMK | 0 | ROX | CIP | 0 |
| PNG | GEN | 0 | ROX | LEV | 0 |
| PNG | TOB | 0 | ROX | NAL | 0 |
| PNG | TET | 0.2 | ROX | TRI | 0 |
| PNG | CLA | 0 | ROX | OXA | 0.12856 |
| PNG | ERY | 0 | ROX | CEF | 0 |
| PNG | CHL | 0 | ROX | NIT | 0 |

**Table S5.** The entire predicted scores ($\gamma=0.6$) were calculated by graph regularization model and synergistic antibiotic combinations were colored red.

| **Drug1** | **Drug2** | **score** | **Drug1** | **Drug2** | **score** |
| --- | --- | --- | --- | --- | --- |
| KAN | AMK | 0 | PNG | CIP | 0 |
| KAN | GEN | 0 | PNG | LEV | 0 |
| KAN | TOB | 0 | PNG | NAL | 0 |
| KAN | TET | 0 | PNG | TRI | 0.23077 |
| KAN | CLA | 0 | PNG | OXA | 0.46154 |
| KAN | ERY | 0 | PNG | CEF | 0.46154 |
| KAN | CHL | 0 | PNG | NIT | 0 |
| KAN | CIP | 0 | ROX | AMK | 0.06207 |
| KAN | LEV | 0 | ROX | GEN | 0.1463 |
| KAN | NAL | 0 | ROX | TOB | 0 |
| KAN | TRI | 0 | ROX | TET | 0.41673 |
| KAN | OXA | 0 | ROX | CLA | 0.35466 |
| KAN | CEF | 0 | ROX | ERY | 0.35466 |
| KAN | NIT | 0 | ROX | CHL | 0.41673 |
| PNG | AMK | 0 | ROX | CIP | 0 |
| PNG | GEN | 0 | ROX | LEV | 0 |
| PNG | TOB | 0 | ROX | NAL | 0 |
| PNG | TET | 0.23077 | ROX | TRI | 0 |
| PNG | CLA | 0 | ROX | OXA | 0.1463 |
| PNG | ERY | 0 | ROX | CEF | 0 |
| PNG | CHL | 0 | ROX | NIT | 0 |

**Table S6.** The entire predicted scores ($\gamma=0.8$) were calculated by graph regularization model and synergistic antibiotic combinations were colored red.

| **Drug1** | **Drug2** | **score** | **Drug1** | **Drug2** | **score** |
| --- | --- | --- | --- | --- | --- |
| KAN | AMK | 0 | PNG | CIP | 0 |
| KAN | GEN | 0 | PNG | LEV | 0 |
| KAN | TOB | 0 | PNG | NAL | 0 |
| KAN | TET | 0 | PNG | TRI | 0.28571 |
| KAN | CLA | 0 | PNG | OXA | 0.57143 |
| KAN | ERY | 0 | PNG | CEF | 0.57143 |
| KAN | CHL | 0 | PNG | NIT | 0 |
| KAN | CIP | 0 | ROX | AMK | 0.1 |
| KAN | LEV | 0 | ROX | GEN | 0.17678 |
| KAN | NAL | 0 | ROX | TOB | 0 |
| KAN | TRI | 0 | ROX | TET | 0.55355 |
| KAN | OXA | 0 | ROX | CLA | 0.45355 |
| KAN | CEF | 0 | ROX | ERY | 0.45355 |
| KAN | NIT | 0 | ROX | CHL | 0.55355 |
| PNG | AMK | 0 | ROX | CIP | 0 |
| PNG | GEN | 0 | ROX | LEV | 0 |
| PNG | TOB | 0 | ROX | NAL | 0 |
| PNG | TET | 0.28571 | ROX | TRI | 0 |
| PNG | CLA | 0 | ROX | OXA | 0.17678 |
| PNG | ERY | 0 | ROX | CEF | 0 |
| PNG | CHL | 0 | ROX | NIT | 0 |

# References

Landrum, G. (2019). RDKit: Open-source cheminformatics from machine learning to chemical registration. *Abstracts of Papers of the American Chemical Society* 258.

Oden, A., and Wedel, H. (1975). Arguments for Fisher's Permutation Test. *The Annals of Statistics* 3(2)**,** 518-520, 513.
